# A Randomized Study on the Effectiveness of Prophylactic Clipping during Endoscopic Resection of Colon Polyps for the Prevention of Delayed Bleeding

**DOI:** 10.1155/2015/490272

**Published:** 2015-02-03

**Authors:** Tatsuya Dokoshi, Mikihiro Fujiya, Kazuyuki Tanaka, Aki Sakatani, Yuhei Inaba, Nobuhiro Ueno, Shin Kashima, Takuma Goto, Junpei Sasajima, Motoya Tominaga, Takahiro Ito, Kentaro Moriichi, Hiroki Tanabe, Katsuya Ikuta, Takaaki Ohtake, Yutaka Kohgo

**Affiliations:** Division of Gastroenterology and Hematology/Oncology, Department of Medicine, Asahikawa Medical University, 2-1-1-1 Midorigaoka-higashi, Asahikawa, Hokkaido 078-8510, Japan

## Abstract

*Backgrounds*. The efficacy of clipping for preventing the delayed bleeding after the removal of colon polyps is still controversial. In order to clarify this efficacy, a randomized controlled study was performed. *Methods*. One hundred and fifty-six patients with colon neoplasms (288 lesions) were enrolled in the study. The patients were randomly divided into two groups: clipping or nonclipping groups using a sealed envelope method before the endoscopic resections. Eight specialists and nine residents were invited to perform this procedure. The risk factors and the rates of delayed bleeding after the endoscopic resections in each group were investigated. *Results*. There were no significant differences in the bleeding rate between the clipping and nonclipping groups, while the length of the procedure was significantly longer and the cost was higher in the clipping group than in the nonclipping group. The rate of bleeding was significantly higher in cases with polyps 2 cm or larger and with a longer procedure time, while none of the other factors affected the bleeding rate. *Conclusions*. This randomized controlled study revealed no significant effect of prophylactic clipping for preventing delayed bleeding after the endoscopic resection of colon polyps.

## 1. Introduction

The most common major complication of endoscopic resection for colon polyps, including polypectomy and endoscopic mucosal resection (EMR), is bleeding [1–7]. The incidence of bleeding after endoscopic resection was reported to be approximately 1% to 6% of polypectomies [8–11]. Most of the bleeding occurs in a delayed fashion from several days to two weeks after the resection [8]. Tumors with a large size and located in the left-sided colon, as well as the use of anticoagulation, are thought to be risk factors for this delayed bleeding [12–21].

A hemostatic clipping device, which was developed by Hachisu et al., has been used for stopping the bleeding during endoscopic resection and has been proposed to be useful to minimize the bleeding during endoscopic resection [22, 23]. Up until now, several large retrospective studies reported contrasting results; however, all showed prophylactic clipping to be beneficial for preventing the delayed bleeding due to endoscopic resections [24–27]. In contrast, the efficacy of prophylactic clipping for preventing delayed bleeding has been investigated by only two prospective studies. One study investigated the bleeding rate of groups treated with and without clipping to prevent the delayed bleeding after the resection of colon polyps in 2003 and found no beneficial effects of the clipping with regard to the delayed bleeding [28]. Another prospective study, which included only cases with pedunculated polyps larger than 10 mm and in which prophylactic clipping was performed before the endoscopic resection, also showed no beneficial effects of the clipping for preventing delayed bleeding [29]. Thus, the efficacy of prophylactic clipping for preventing delayed bleeding is still controversial, and the necessity to perform prophylactic clipping after endoscopic resection has up to now been empirically judged by individual physicians or institutes.

The present randomized controlled study investigated the rate of delayed bleeding and the additional costs associated with endoscopic resections to clarify the efficacy of prophylactic clipping for preventing delayed bleeding.

## 2. Methods

### 2.1. Patients

This study was registered with the University Hospital Medical Information Network (ID UMIN000013856 number; R000016161). Written informed consent was obtained from all patients enrolled, and the study was approved by the Institutional Review Board of Asahikawa Medical University (number 1404). One hundred and fifty-six patients who had colon neoplasms and underwent endoscopic resection, including polypectomy and EMR, at Asahikawa Medical University Hospital between October 24, 2011, and April 3, 2014, were enrolled in this prospective study. The ratio of males to females was 108 : 51, and the average age of the patients was 67.5 ± 10.1 years.

### 2.2. Endoscopic Procedures

The patients were randomly divided into two groups, a clipping group and a nonclipping group, using a sealed envelope method before the endoscopic resection. A total of 288 lesions, which were indicated for endoscopic resection, were detected in the enrolled patients. Of these 288 lesions, 54 and 234 were removed by polypectomy and EMR, respectively. All polyps were resected using an ERBE ICC200 (Endocut mode, effect 3, 120W) or ERBE VIO300D device (Endocut Q mode, effect 2, duration 2, interval 4). When an EMR procedure was used for the removal, a submucosal injection of saline solution with a small amount of indigo carmine was performed, and then the polyps were removed. In the clipping group, the sites of endoscopic resection were completely closed by clipping, with gaps of less than 1 cm between the clips, using Resolution HX-610-135 clips (Olympus Co., Ltd., Tokyo, Japan). The procedure time was measured from saline injection to the removal of the lesion in the nonclipping group or from the saline injection to the finalization of the clipping in the clipped group.

Eight specialists and nine residents were invited to perform endoscopic resections. Specialists were defined as physicians who had performed more than 100 endoscopic resections. Residents were defined as physicians who had experienced fewer than 100 endoscopic resections. Each participant independently removed the lesions and performed the clipping. The procedural cost of the clipping was calculated by adding the cost of the devices and the staff, including endoscopists and nurses.

### 2.3. Definition of Bleeding

After the endoscopic resection, the patients were hospitalized for one day. All patients came to our hospital within four weeks after discharge. If delayed bleeding occurred, the patients immediately came to our hospital and underwent emergency colonoscopy. Postpolypectomy or post-EMR bleeding was defined as a case of obvious anal bleeding with a more than 2 g/dL decrease in the blood hemoglobin concentration and/or the development of hypovolemic shock. The bleeding points of the ulceration formed by polypectomy or EMR were identified by colonoscopy in all cases with delayed bleeding.

### 2.4. Statistical Analysis

In our prospective study, Chi-square test with Yates' correction and Mann-Whitney  *U* test were applied for the statistical analysis of the relationships between the bleeding rate and the patients' demographic data, the characteristics of the lesions, the endoscopists' experience, and the performance of clipping. A value of *P* < 0.05 was considered to be statistically significant.

## 3. Results

### 3.1. Association of the Rate of Delayed Bleeding with the Patients' Demographic Data, the Characteristics of the Lesions, and the Experience of the Endoscopists

The number of lesions in male and female patients was 208 and 80, respectively. The ages of the patients ranged from 29 to 85 years. Eight and 38 lesions were found in the patients who were taking anticoagulant and antiplatelet drugs, respectively, which were stopped three to seven days before the endoscopic resection. Of the 288 lesions, 184 lesions were less than 10 mm in diameter, 90 were 10 to 20 in diameter, and 14 were 20 mm in diameter or larger. Nineteen, 54, 70, 25, 96, and 27 lesions were located at the cecum, ascending colon, transverse colon, descending colon, sigmoid colon, and rectum, respectively. The histological diagnosis of these lesions included 273 adenomas and 15 carcinomas* in situ*.

One hundred and seventy-seven lesions were removed by experts and 111 were removed by less-experienced endoscopists. There were no significant differences between the bleeding rate based on the patients' demographic data, including their gender, age, or the administration of anticoagulant and antiplatelet drugs. Among the factors associated with the lesions, the rate of bleeding was significantly higher in the cases with lesions 2 cm or larger in size. A longer procedure time, which was proportional to the size of the lesion, was associated with a risk for delayed bleeding. However, the location of the lesions, endoscopic procedure, morphology of the lesion, histological diagnosis, and the experience of the endoscopists did not affect the rate of delayed bleeding (Table 1).

### 3.2. The Rate of Delayed Bleeding and Procedural Cost in the Clipping and Nonclipping Groups

**Figure 1:**
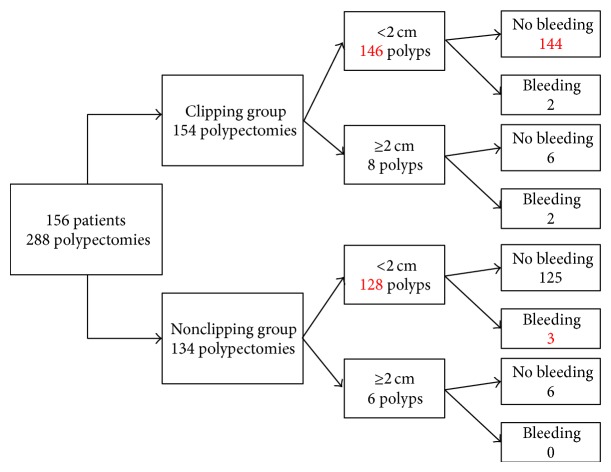
The tumor size and the number of lesions with delayed bleeding.

One hundred and fifty-four lesions were part of the clipping group and 134 were in the nonclipping group. There were no significant differences between the groups in terms of the gender, age, tumor location, morphology or size, the administration of anticoagulants and antiplatelet agents, the endoscopic procedure, obvious vessels in the ulceration after endoscopic resection, or the experience of the endoscopists performing the resections. The length of the procedure was significantly longer in the clipping group (528 ± 559 seconds) than in the nonclipping group (281 ± 263 seconds). In the clipping group, four of the 154 lesions exhibited delayed bleeding after endoscopic resection, while three of the 134 lesions exhibited bleeding in the nonclipping group(Table 2). Of the 14 lesions 2 cm or larger in size, two of eight and zero of six lesions exhibited bleeding in the clipping and nonclipping groups, respectively. There was no significant difference in the bleeding rate between the clipping and nonclipping groups, regardless of the size of the lesions (Figure 1).

The cost of the participants was 157 yen/minute, and the average difference in the length of the procedure was 242 seconds. Therefore, the additional cost for the participants in each case in the clipping group was 633 yen. The cost of a clip was 1,000 yen, and the average number of clips used in one procedure was 2.16. Therefore, the total cost of the clips used in each case was 2,160 yen. The additional cost of the clipping group was therefore 2793 yen (about 28 U.S. dollars) per lesion.

## 4. Discussion

This randomized controlled study compared the rates of delayed bleeding after endoscopic resection between the clipping and nonclipping groups and revealed that prophylactic clipping had no significant effect in preventing the occurrence of delayed bleeding, although the performance of such prophylactic clipping was a time-consuming procedure that cost about 2,553 yen (about 25 U.S. dollars) extra for each case. To date, the necessity of the prophylactic clipping after endoscopic resection has been empirically judged by individual physicians or institutes, because the indications for the procedure have not been established. Shioji et al. proposed in their prospective study that the prophylactic clipping was associated with no beneficial effect for preventing delayed bleeding [28]. However, that prospective study was conducted more than 10 years ago, and new clipping devices, including various-sized clips, rotator clips, and high frequency generators, have been developed during these 10 years. The present study also showed no beneficial effects of the prophylactic clipping even though we used the latest devices. Quintanilla et al. investigated the efficacy of a new clipping procedure, in which the prophylactic clipping was performed for the stalk of a pedunculated polyp before endoscopic resection and showed no beneficial effect of the procedure for preventing delayed bleeding [29]. In contrast, two retrospective studies showed that prophylactic clipping was an effective procedure for preventing the delayed bleeding due to endoscopic resection [25, 26]. These two studies included only large lesions (≥2 cm), while Shioji's study [28] and our present study included a small number of large tumors. Taken together, prophylactic clipping is considered to be less useful for preventing the occurrence of delayed bleeding after endoscopic resections for small lesions, but it might be effective for large lesions (measuring 2 cm or more in size).

The present study showed the procedure time to be associated with the rate of delayed bleeding, regardless of whether or not prophylactic clipping had been performed. Technical difficulties, including the site at which the lesion is insufficiently observed and it was difficult to manipulate the scope, as well as the size of the lesion, are thought to be associated with the procedural time. Further analyses concerning the procedure time and the rate of delayed bleeding, including the reasons for a longer procedure time, are expected to support the importance of the technical aspects for the prevention of delayed bleeding after endoscopic resection.

It has been reported that treatment with anticoagulants was a risk factor for bleeding after endoscopic resection [21]. A recent decision analysis proposed that using a prophylactic clip after polypectomy was a cost-effective strategy for patients who were receiving antiplatelet or anticoagulation therapy, but the reference case was a 50-year-old patient with a 1.0 to 1.5 cm polyp [30]. However, the present study showed that the treatment with anticoagulants did not affect the rate of delayed bleeding, regardless of whether clipping was performed, because the treatment of anticoagulants was stopped three to seven days before the endoscopic resection in this study. This suggests that the prophylactic clipping is not effective to prevent delayed bleeding after endoscopic resection when the anticoagulant treatment is appropriately stopped before the procedure.

We also investigated whether the efficacy of the prophylactic clipping was associated with the experience of the endoscopist. However, the rate of the delayed bleeding after endoscopic resections performed by specialists (3/177; 1.7%) was not significantly lower than that performed by less-experienced endoscopists (4/111; 3.7%). This suggests that the endoscopic experience alone cannot sufficiently decrease the rate of delayed bleeding. A strategy for preventing delayed bleeding should be established based on the precise evaluation of risk factors, including the tumor size and the use of new devices using an endoloop, as well as hemostatic forceps.

The present study is associated with several potential limitations. One of the limitations is the small number of the enrolled patients. However, the rate of the delayed bleeding in each group was almost the same, suggesting that no advantage of the prophylactic clips would likely be observed even if the study included a large number of patients. Second, the present study included only a small number of large (2 cm or larger) polyps. It has been known that a larger polyp size is a risk factor for delayed bleeding after endoscopic resections. Therefore, the present study may not provide an accurate assessment of the efficacy of prophylactic clipping to prevent the occurrence of delayed bleeding in larger sized polyps, and the present results may be applicable only for small-sized polyps. Third, the study was conducted at a single educational hospital. Further multicenter studies with larger numbers of patients and various sizes of polyps will be warranted to determine the best strategy for preventing delayed bleeding after endoscopic resections of colon polyps.

The size of the lesions and the number of lesions with delayed bleeding after the endoscopic resections were described in the flow chart. No significant difference of the bleeding rate was observed between the clipping and nonclipping groups, regardless of the size of the lesions.

## Figures and Tables

**Table 1:** The characteristics of the patients and lesions and the endoscopic factors in the clipping and nonclipping groups.

		Total	Bleeding	No bleeding	*P* value
Numbers of lesions in male and female patients	Male	208	6	202	N.S.
	Female	80	1	79	

Age (mean ± SE)		67.4 ± 10	62.4 ± 13	67.5 ± 10	N.S.

Anticoagulant drugs	+	8	0	8	N.S.
	−	280	7	273	

Antiplatelet drugs	+	38	2	36	N.S.
	−	250	5	245	

Location	Rectum	27	1	26	N.S.
	Others	261	6	255	
	Cecum	19	0	19	
	Ascending	54	1	53	
	Transvers	70	3	67	
	Descending	25	0	25	
	Sigmoid	96	2	94	

Morphology	Pedunculated	41	1	40	N.S.
	Sessile and flat	247	6	241	

Size (cm)	<2	274	5	269	
	≥2	14	2	12	<0.01

Pathology	Adenoma	273	5	268	N.S.
	Carcinoma	15	2	13	

Method	EMR	234	5	229	N.S.
	Polypectomy	54	2	52	

Exposed vessel	+	7	1	6	N.S.
	−	281	6	275	

Length of procedure (mean ± SE)		413 ± 463	937 ± 1623	396 ± 360	<0.01

Specialists	+	177	3	174	N.S.

Less-experienced endoscopists	−	111	4	107

N.S.: not significant.

EMR: endoscopic mucosal resection.

Exposed vessel: obvious vessels in the ulceration after endoscopic resection.

**Table 2:** The characteristics of the patients and lesions and the endoscopic resection-associated factors in the clipping and nonclipping groups.

		Clipping	Nonclipping	*P* value
Numbers of lesions in male and female patients	Male	109	99	N.S.
	Female	45	35	

Age (mean ± SE)		67.1 ± 8	67.8 ± 11	N.S.

Location	Rectum	11	16	N.S.
	Others	143	118	

Morphology	Pedunculated	28	13	N.S.
	Sessile and flat	127	122	

Size (cm)	<1	98	86	N.S.
	≥1, <2	48	42	N.S.
	≥2	8	6	N.S.

Anticoagulant drugs	+	6	2	N.S.
	−	148	132	

Antiplatelet drugs	+	19	19	N.S.
	−	135	115	

Treatment method	EMR	131	103	N.S.
	Polypectomy	23	31	

Exposed vessel	+	5	2	N.S.
	−	149	132	

Length of procedure (second; mean ± SE)		528 ± 559	281 ± 263	<0.01

Specialists Less-experienced endoscopists	+	87	90	N.S.
	−	68	43	

Bleeding	+	4	3	N.S.
	−	150	131	

N.S.: not significant.

EMR: endoscopic mucosal resection.

Exposed vessel: obvious vessels in the ulceration after endoscopic resection.
